# The fluctuation of pig prices and the identification of major drivers in China

**DOI:** 10.1371/journal.pone.0313982

**Published:** 2024-11-22

**Authors:** Junguo Hua, Jing Ding, Yufan Chen, Lulu Kang, Haiying Zhang, Junhua Zhang

**Affiliations:** 1 College of Economics and Management, Henan Agricultural University, Zhengzhou, Henan, China; 2 College of Information Science and Technology, Zhengzhou Normal University, Zhengzhou, Henan, China; 3 College of Economics and Management, China Agricultural University, Beijing, China; Obafemi Awolowo University, NIGERIA

## Abstract

In recent years, the domestic live pig price has risen and fallen rapidly and fluctuated frequently, which has greatly impacted the live pig industry. The level of price volatility in the pig market has served as a significant indication of the progress of the agricultural sector. Identifying the dominant factors affecting the fluctuation of pig prices has become more important. Based on the monthly data of the pig industry from February 2009 to December 2022, this paper constructs an index system of influencing factors of pig price from four aspects: uncertain impact, supply factors, demand factors, and macro-environment factors. By using the transfer model of the Markov regime (MS-VAR), we obtained the probability plot of zone transition for pig price volatility, the impulse response effect diagram of factors affecting pig price, and the cumulated impulse response effect diagram of factors affecting pig price, and analyze the reasons for the ups and downs of pig price according to the above results. The findings indicate prominent features of zone transition in the price fluctuation of China’s pig market. From 2017 to 2022, the domestic pig price frequently switches between rising and falling zones, and the "falling pig price stage" and "rising pig price stage" in the non-stationary state last for a relatively short and discontinuous period. There is little probability that the price of live pigs will directly change from rising to falling, and there will be a smooth buffer stage in the price rise and fall process. Among the factors that affect the fluctuation of live pig prices, the dominant factor of frequent and large fluctuation of pig prices is the pig epidemic situation in external factors. Among the internal influencing factors, the changes in farming costs have the greatest significant influence on the fluctuation of pig prices. These results provide a decision-making reference for legislators to carry out epidemic risk prevention and control better, stabilize the market pig price, and provide empirical evidence for market participants to accurately avoid price risks through multiple channels and ways and ensure stable profitability.

## 1. Introduction

Pigs are one of the leading livestock products in the world. The price level in the pig market has served as a significant indicator of the progress of the livestock sector, and it is also a key factor affecting farmers’ production decisions, consumers’ purchase behavior, and the overall operation of related industrial chains. With the emergence of African swine flu in 2018, the country’s pig business has entered a new phase of the “pig cycle". This "pig cycle" round is superimposed with environmental protection and limited production, and the international food price rises, and the amplitude fluctuates sharply. The price fluctuates at a high level in the early stage and continues to fall in the later stage. Therefore, the domestic pig industry has been dramatically affected. In recent years, the marketization of pigs has gradually matured, and the influencing factors of live pig price fluctuation are more diversified, which are not only limited to the relationship between supply and demand but also affected by many kinds of factors such as production cost, policy, disease epidemic situation, and substitute price.

Considerable debate has arisen over the attributes and determinants of pig price volatility. Pig prices see two sorts of changes: regular fluctuations and irregular fluctuations. Irregular variations are the primary issue hindering the pig business’s healthy and steady growth [1]. As a result of the intricate nature of the market, pig prices are influenced by several unpredictable variables, leading to frequent fluctuations and displaying distinct non-linear fluctuation patterns at various time intervals [2–4]. Based on the study conducted by previous researchers, the variables that influence pig pricing may be broadly categorized into internal components and external aspects. The cumulative impact of several variables eventually affects the trajectory of pig prices.

Internally, the price variations of pigs are influenced mainly by supply and demand variables. Any unexpected changes in supply or demand may lead to swings in the price of livestock products [5–9]. At the supply level, the growth cycle of pigs from piglets to farrowing is lengthy, which poses a challenge for producers to respond promptly to market conditions. This extended growth cycle has become a significant issue in regulating pig prices [10, 11]. Various factors contribute to fluctuations in pig prices, including piglet prices, the number of breeding sows, producer expectations of pork prices, and the diverse decisions and behaviors of different producers based on market information [12–16]. Additionally, the rising costs of feed, labor, and veterinary health care during the breeding process contribute to an increase in the price of pork [8, 17, 18]. With the recovery of pig production capacity in 2020, there has been a drop in the number of retail homes engaged in pig farming. Consequently, pig farming has been increasingly shifting towards larger-scale operations. The implementation of large-scale agriculture has a notable stabilizing impact on the volatility of pig prices and has a substantial regional spillover effect [19, 20]. At the demand level, Consumer behavior significantly influences short-term fluctuations in pork prices [21–24]. Additionally, the market for livestock and poultry products is interconnected, with price spillover effects observed between different links in the pig industry chain [25, 26]. There is a wide range of pork substitutes, and price spillover effects are evident between pork and other livestock and poultry products. Consequently, simultaneous changes in the prices of various pork substitutes can lead to significant fluctuations in pork prices [27–29].

Regarding external factors, the advancement of economic globalization and the growth of international economic connections will intensify the imbalance between pig supply and demand. This will result in increased volatility in pig prices and pose liquidity risk and systemic risk to the entire pig supply chain [30–32]. Episodic variables such as outbreaks of pig illnesses, natural catastrophes, wars, trade frictions, and other unforeseen circumstances may lead to short-term swings in pig prices or the extension. [33–35]. The intermittent and unpredictable shocks lack enduring and self-regulating mechanisms, resulting in a long-term influence on pig prices. Prior research has consistently demonstrated that significant epidemics, such as those affecting pigs, not only emphasize the discrepancy between the supply and demand of pig markets, leading to extensive and enduring fluctuations in pig prices, but also have a subsequent impact on the prices of interconnected products within the pig industry chain [36, 37]. African swine fever’s disastrous outbreak in 2018 led to a severe supply shortage in the pig market, a significant increase in pig market prices, and a severe supply-demand imbalance in the meat market [38–40]. The direction and size of the shock of the pig epidemic varied at different points in time during various periods, and the duration of the shock was long [41, 42]. Additionally, researchers have shown that outbreaks of pig diseases may worsen the imbalance between supply and demand, leading to substantial and enduring fluctuations in the price of pork. Furthermore, these epidemics can cause incalculable financial losses for pig farming businesses. The growing market economy in China has led to a more significant influence of macroeconomic factors on the fluctuation of pig prices [43–46].

The money supply has a non-neutral effect on pig pricing, and the influence and transmission of international pig prices to China’s pork retail market have steadily become apparent [47–49]. According to Guo Fan et al., economic policy uncertainty significantly influences domestic agricultural commodity prices. This influence is observed through various channels, including supply and demand, financial, and international markets. Economic policy uncertainty impacts livestock meat products more than other agricultural products [50–52]. Shi Zizhong et al. [53] and Guo Jingchi et al. [54] demonstrated that economic policy uncertainty shocks have a significant and lasting impact on the price of pork, particularly within the livestock industry. These shocks also affect the price of the entire pig industry chain, which experiences significant periodic fluctuations. Effective and significant policy regulation will have a crucial impact on the patterns of pig prices [55]. The unpredictable disturbances have an immediate impact on the typical pattern of pig prices in the short term. They will also disturb the regular cycle of pork prices in the long term, causing abnormal fluctuations in pig prices. This, in turn, alters the consumption habits of urban and rural residents and leads to hasty decision-making by farmers. Consequently, it significantly affects the overall healthy growth of the industry.

In summary, fluctuations in pig prices are influenced by several factors, including supply and demand dynamics, macroeconomic conditions, government regulations, and natural calamities. Both domestically and internationally, researchers have provided several answers based on diverse research perspectives on the fluctuation of pig prices, including their causes and the elements that influence them. Most experts agree that the primary cause of the volatile short-term pig prices is the external uncertainty shock experienced by the pig market. Additionally, the recurring swine pandemic is considered the critical factor contributing to establishing the "pig cycle". However, these studies have certain limitations. Firstly, the research perspective tends to be narrow, making it challenging to thoroughly analyze the factors contributing to the fluctuations in pig prices. The definition of the dominant factors driving these price fluctuations is not sufficiently clear. Secondly, the causes of pig price fluctuations are intricate, involving numerous influencing factors. The crucial factors that significantly impact pig price fluctuations have not been identified during efforts to mitigate these fluctuations and manage price risks.

The innovation of this study primarily manifests in the following aspects: Firstly, by systematically reviewing the factors influencing pig price fluctuations and applying factor analysis, a comprehensive index system encompassing various factors affecting pig price fluctuations has been constructed from multiple dimensions, striving to comprehensively and exhaustively cover all relevant factors. Secondly, when building the empirical model, this paper fully considers the time-varying characteristics of time series. Traditional time series models often preset the coefficients and the variance of random disturbance terms as non-time-varying. However, vital economic variables such as financial structures and institutional policies frequently exhibit significant structural changes over time. Given this, this paper adopts the Markov-Switching Vector Autoregressive (MS-VAR) model for empirical analysis, as this method can more effectively capture and depict the dynamic characteristics of various variables over time. Utilizing the OxMetrics software, we ultimately constructed impulse response plots for the factors influencing pig price fluctuations. These plots enable a relatively straightforward and precise determination of the dominant factors behind significant pig price movements and the maximum cumulative impact values and timing of these dominant factors. This comprehensive and in-depth analytical approach transcends traditional time series analysis, enhancing the accuracy of the research and improving its readability and practicality. It provides new empirical analysis tools and insights for subsequent studies, significantly advancing and expanding the existing literature. Furthermore, it offers a more scientific basis and robust support for the sustainable development of the pig industry.

Due to the influence of many factors, pig prices often fluctuates violently in the short and long term, which results in frequent and significant fluctuations in both short and long terms; this phenomenon not only poses considerable challenges to accurate market trend prediction but also severely impedes the stable development of the pig industry. Therefore, an in-depth exploration of the changing patterns of the dominant factors behind pig price fluctuations and their far-reaching impacts is particularly crucial and urgent.

This study aims to comprehensively dissect the operational mechanisms of the pig market by systematically identifying and quantifying the key factors influencing pig prices. It aims to reveal how these factors interact and collectively drive dynamic price changes. This process enhances our understanding of the pig market’s complexity and enriches the research literature on pig price fluctuation analysis. Furthermore, it provides scientific evidence and insights for agricultural practitioners, market analysts, and policymakers.

From a practical perspective, identifying the dominant factors and their impact on pig price fluctuations offers direct guidance for mitigating price risks and optimizing production decisions. Agricultural enterprises and farmers can adjust production plans and rationally arrange the scale of breeding based on these research findings to cope with market uncertainties and risks. More profoundly, the findings of this study can provide robust data support and theoretical foundations for agricultural policymakers, enabling them to formulate more precise and practical policy recommendations. These policies aim to stabilize pig market prices, promote sustainable industry development, and ensure stable incomes for farmers, thereby driving the prosperity and progress of the entire agricultural economy.

In summary, studying the changes in dominant factors of pig price fluctuations and their impact effects enriches the existing literature on the fluctuation of live pig prices, provides a new method, and provides valuable insights into the complex dynamics of the fluctuation of live pig prices. It is a vital component of deepening theoretical research on the pig market and a key factor in guiding practice and promoting the healthy development of the pig industry.

## 2. Methodology

Based on the model established by Melitz and Ottaviano [56], this paper constructs a theoretical framework for pig prices and the main influencing factors and then discusses the micro-influence mechanism of pig price fluctuations to provide a theoretical basis for the empirical test later.

### 2.1 Demand factors

Pork is the primary source of protein for China’s residents, and its consumption has always occupied a large proportion of meat consumption; compared with pork supply, the demand for pork is rigid and inelastic, and at the level of consumer demand is mainly the price of substitutes, and consumer behaviors affects the fluctuation of pig prices.

(1) Price of Alternatives

Presently, China’s pork consumption presents a diversified development trend; consumers pay more attention to the health and nutritional value of the product, and the demand for medium and high-end pork products is rising year by year. However, the domestic pig industry generally exists in the pursuit of high yield and neglects the guarantee of product quality and taste. At the same time, pig diseases have also brought specific safety hazards to the quality of pork products in China. Under the effect of a series of factors, pork consumption has decreased while the demand for its substitutes has increased. In addition, the price changes of pig substitutes will affect the demand for pigs to a certain extent. If the price of its substitutes increases, it will reduce the residents’ demand for substitutes, which will increase pork consumption and lead to an increase in the pig prices. As people’s living standards continue to rise and meat products diversify, this leads to more substitutes, the substitution effect increases, and the impact of substitutes becomes more complex.

(2) Consumer Behaviors

Pork is a typical product with an elasticity of demand greater than 0. An increase in the population’s income level increases the demand for pork. Thus, the price of pork, but the extent of this effect tends to narrow with the improvement in the standard of living of the population and the growth of income. High-income people have the weakest pork consumption, followed by middle-income people, and low-income people have the greatest pork consumption. In addition, animal diseases, policy introduction, and changes in the economy can change consumers’ future expectations and thus affect consumer behavior. These factors can lead to changes in the demand for pork consumption and thus affect price fluctuations.

Draw lessons from the consumer utility function set in the study of Melitz and Ottaviano [56], such as Formula (1):

$$U=q_{0}^{c}+\alpha \int_{i\in \Omega }q_{i}^{c}di-\frac{1}{2}\gamma {\int_{i\in \Omega }\left(q_{i}^{c}\right)}^{2}di-\frac{1}{2}\eta {\left(\int_{i\in \Omega }q_{i}^{c}di\right)}^{2}$$ (1)

This study assumes that market consumers obey the following quasi-linear preferences:

$$U=q_{0}^{c}+\alpha \int_{i\in \Omega }d_{i}q_{i}^{c}di-\frac{1}{2}\gamma {\int_{i\in \Omega }\left(d_{i}q_{i}^{c}\right)}^{2}di-\frac{1}{2}\eta {\left(\int_{i\in \Omega }d_{i}q_{i}^{c}di\right)}^{2}$$ (2)

Eq (2), $q_{0}^{c}$ and represents the quantity of market consumers’ consumption of cold pork and pork alternatives, respectively, parameters *α* and *η* denote the elasticity of substitution between both pork and pork alternatives, respectively, and indicates the degree of mutual substitution between substitutes of different kinds of pork alternatives. Ω denotes the set of heterogeneous commodity categories, and *d*_*i*_ portrays heterogeneity in the degree of substitution between different types of alternatives, and the above parameters are positive. Given the budget constraint and considering the utility maximization problem of consumers in the market, the function of aggregate demand in the pig market can be found as:

$$Q=Lq_{i}^{c}=\frac{L}{\gamma d_{i}}\left(\frac{\gamma \alpha +\eta \int_{i\in \Omega }\frac{p_{i}}{d_{i}}d_{i}}{\gamma +N\eta }-\frac{p_{i}}{d_{i}}\right)$$ (3)

In Eq (3), *N* denotes the number of types of pork substitutes and *L* denotes the market size. From Eq (3), it can be seen that in the case of a certain market size, the types of pork alternatives *N*, the degree of substitution between alternatives *γ* and the degree of heterogeneity of alternatives in the degree of substitution *d*_*i*_ three related indicators are negatively correlated with the total pork market demand *Q*. The larger its value, the smaller the total pork market demand *Q*, which in turn causes the price of pigs to decrease. On the contrary, the smaller the value of the pork alternatives-related indexes, the higher the price of pigs will be pushed up. Changes in the substitution utility of substitutes for pork in the meat market will lead to fluctuations in the pig price.

### 2.2 Supply factors

Supply factors constitute a significant factor influencing the volatility of pig prices that play a central part in the transmission system of pig price fluctuations [57, 58]. According to previous studies and combined with the reality of careful consideration, the influence of factors affecting the price of pigs mainly include the following aspects:

(1) The Cost of Inputs

Whether a pig farming enterprise can maximize the relative returns from the production process is the primary basis for its production decisions, so the cost of farming is an essential influence on pork supply. In theory, pig production factor prices and prices show the same trend of change: rising (falling) prices of production materials cause pork prices to rise (fall). The cost of inputs in the process of pig farming mainly includes fixed costs and variable costs, fixed costs such as plants and instruments that cannot be adjusted in the short term once they are put into use, and variable costs include feed costs, piglet fees, and other costs. Farmers are only the recipients of their prices. Pig feed costs account for 60% of the total cost of pig farming in China, and with the expansion of the scale of pig farming, the proportion of pig farming costs will be reduced. The increase in grain prices in recent years has led to a rise in feed costs, which is transmitted to an increase in pig farming costs, ultimately leading to an increase in pig and pork prices. It takes 5–6 months from the purchase of piglets to farrowing, and the inconsistency between piglet prices and the profitability of farrowed pigs will increase the uncertainty of farmers’ earnings and the volatility of pig market supply in the later period. The cost of piglets accounts for about 20% of the total cost of pig farming, another major factor affecting the cost and price of pork.

(2) Stock of Breeding Swine

The availability of pork is contingent upon the quantity of pigs received, and the amount of breeding pigs will directly impact the pigs’ production potential. The number of piglets born is contingent upon the number of pigs breeding over the 12 months before the present farrowing cycle. This implies that, in the absence of more breeding pigs to augment the pig population, the supply of pigs will be restricted.

Therefore, the supply of pigs is closely related to the holding of breeding sows with a one-year lag, and improving the productivity of holding breeding sows can reduce the unit cost of production and increase the adequate supply of pigs. Because of the “pig cycle”, farmers the pig prices when the price rises to replenish fences, the culling of sows in times of declining pig prices due to going after profit, and smallholder awareness can exacerbate the volatility of pork prices in the pig market.

(3) Uncertainty Shocks

Pig diseases are difficult to predict and have a high degree of uncertainty. First domestic epidemic of African swine fever in 2018, the disease in pigs will spread to the surrounding area horizontally or vertically, leading to a rise in the death rate of pigs, an increase in the number of culls, and a consequent plunge in the number of pigs stocked in and out of pens, and an increase in the supply gap. The health condition of breeding sows is closely correlated with the likelihood of pig illness. Outbreaks of disease may result in the mortality of a substantial quantity of breeding sows, thereby causing a significant decrease in pig production capability. Following the pandemic, there may be a conflict between the quick increase in pork consumption and the slower recovery of pig production capacity. This might result in an imbalance between the supply and demand of pigs, leading to more significant volatility in pig prices. Due to the African swine disease pandemic, pig prices will see more pronounced changes compared to previous "pig cycles". This will result in a broader price difference between the highest and lowest prices, leading to a somewhat longer cycle of pig price variations.

China’s livestock and poultry products market is open to the outside world [59]. The higher the economic policy uncertainty index, the more unstable the general market environment, including the pig market, and the less optimistic the prospects for economic development [60]. The influence of economic policy uncertainty on pig prices mainly manifests in two aspects: its immediate effect and its delayed impact in the current period are primarily caused by market panic, in which the wholesalers with substantial market power to judge the economic situation at the right time to choose to add to the pricing, and the consumer as the price of the recipient, can only adjust their consumption structure and thus promote the changes in market prices; in the lagged period of impact is mainly manifested in the effects on the future changes in production. Agricultural manufacturers adjust the price of agricultural production materials according to changes in the economic market situation, which affects the cost of pig production, after which producers adapt their production plans due to the impact of the economic environment and fluctuations in costs. Agribusiness manufacturers and producers work together from the supply side to influence future pig market pricing.

As mentioned earlier, it is assumed that the uncertainty shock to the pig industry is mainly manifested in the two aspects of pig epidemics and global economic policy uncertainty, and both of them jointly affect the supply of the pig industry. In this case, the factor inputs of pig farming are categorized, and the marginal output of the factors can be used to measure productivity. Assuming that each firm entering the pig industry pays fixed costs of *f*_*e*_, the initial productivity level of the firm is *φ*_*i*_. Assuming that each firm faces an uncertainty shock index of *uncind*_*i*_, which measures the extent to which the combined capacity of the pig firms, such as breeding sows and pigs, is impaired. Set the value of the unfavorable uncertainty shock index as positive; the more significant the value is, the larger the decrease in pig inventory is; the value of the favorable uncertainty shock index is negative; the larger the value is, the larger the increase in pig inventory is. This further gives the actual productivity of firms adjusted by the uncertainty shock index as (1 − *uncind*_*i*_)*φ*_*i*_. Since different enterprises have different degrees of large-scale farming, there are differences in input costs, management modes, animal epidemic prevention measures, etc. The size of the uncertainty shock index *uncind*_*i*_ is related to the enterprise’s degree of large-scale farming, *I*_*i*_. Drawing on Hallak and Sivadasan [61], Gervais [62], assuming that the firm’s cost function is *f*(*I*_*i*_) and *f*′(*I*_*i*_) > 0, *f*″(*I*_*i*_) > 0, and that the firm wants to increase its level of scale farming, then it needs to pay more fixed costs to achieve a higher level of scale, Then the stopping point of the business is:

$$C_{D}=P_{\text{max}}=\frac{\gamma \alpha +\eta \int_{i\in \Omega }\frac{p_{i}}{d_{i}}di}{\gamma +N\eta }$$ (4)

Under the condition of monopolistic competition, according to the condition of profit maximization of the enterprise, it can be found that the pricing of pigs by the pig enterprise in equilibrium and the profit gained are:

$$P(unc.ind,\varphi )=\frac{1}{2}(C_{D}-\frac{1}{\varphi (1-unc.ind)})$$ (5)

$$\pi (unc.ind,\varphi ,I)=\frac{L}{4\gamma }{\left(C_{D}-\frac{1}{\varphi (1-unc.ind)}\right)}^{2}-f(I)$$ (6)

From formulas (5) and (6) can be seen, the case of other factors must be the pig market by unfavorable uncertainty shock, the greater the degree of impact, the enterprise to ensure profits and reduce losses, will be as early as possible to minimize the market pricing of pigs to ensure that pigs can be sold, and the profit obtained by it will be relatively lower than the typical situation; Similarly, when the price of pigs by the favorable uncertainty shock (for example, accelerated economic growth), the market price of pigs will be followed by the price of pigs, so that pig enterprises to obtain the profits of a relatively high.

### 2.3 The formation of pig price volatility

In contrast, the volatility of pig prices is mainly attributed to the disparity between supply and demand. On the supply side, the short-term elements that most influence the volatility of pig prices are the quantity and average weight of pigs sold, import volume, and national reserve sales. The long-term factors essentially consist of the pig-raising cycle and the cost of pig farming.

On the demand side, consumer purchasing power is affected by external uncertainty shocks, including income levels, population size and structure, consumption seasonality and substitute prices, epidemics, and economic policy uncertainty. Because the timing and extent of the shocks are difficult to predict, the impact on pig prices can be more dramatic, leading to abnormal fluctuations in pig prices that affect both supply and demand.

**Fig 1:**
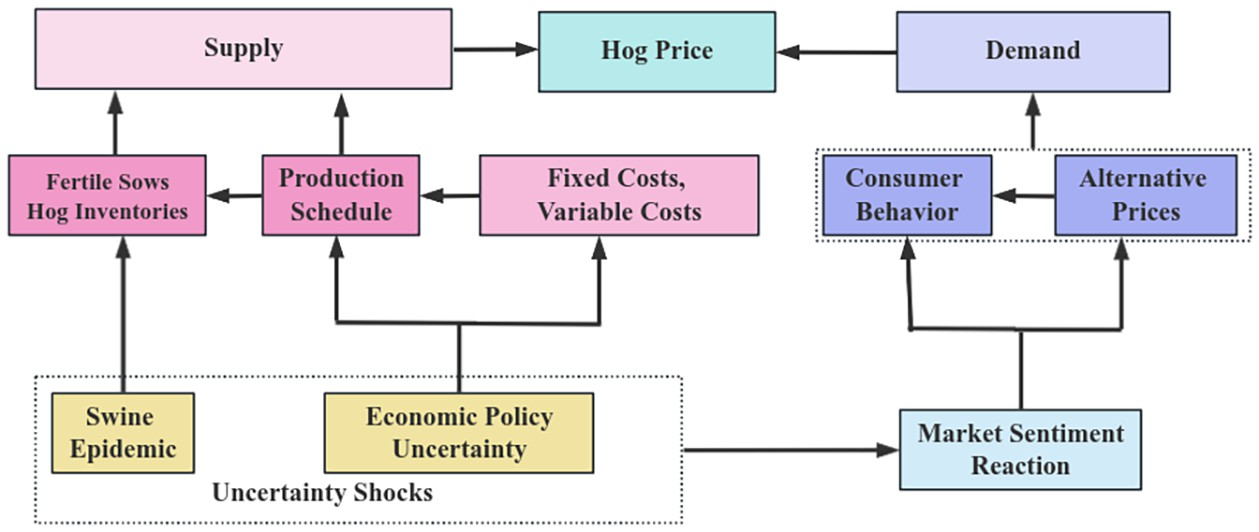
Path diagram of pig price volatility formation.

According to the above analysis, the formation path diagram of pig price fluctuation can be clearly presented in Fig 1.

## 3. Data and methods

### 3.1 Econometric strategies

According to previous studies, the relationship between pig price and various influencing factors is not a simple linear relationship but needs to be discussed in depth from a nonlinear perspective. In this context, the transfer model of Markov regime (MS-VAR) shows its unique applicability. At first, the model was widely used to study economic cycles to capture the transition of economic states and the dynamic characteristics in different States. Similarly, in the field of pig price research, the market is affected by multiple factors, such as epidemic outbreaks, policy adjustments, changes in supply and demand, etc., which often lead to significant fluctuations and asymmetries in pig prices in different periods.

Compared to the traditional VAR model with fixed parameters, the MS-VAR model can estimate regression parameters under different regimes and fully accommodate structural changes that may occur in the generation process of time series data. This characteristic makes the MS-VAR model particularly suitable for analyzing market phenomena such as pig prices, which exhibit distinct phased characteristics and are influenced by complex factors. Through this model, one can identify the transition probabilities of pig price among different regimes (e.g., rising, falling, and stable periods) and the impulse responses and cumulated responses of various influencing factors to pig price. This allows a deeper understanding of the nonlinear mechanisms and driving forces behind pig price fluctuations. Therefore, when exploring the complex relationship between pig prices and their influencing factors, the MS-VAR model provides more precise fitting results and reveals more detailed information about market structure and dynamic behavior, making it suitable for the empirical research conducted in this study.

The MS-VAR model is a modification of the classic vector autoregressive model. In this model, the coefficients produced from the observable time-series data-generating process are influenced by an unobservable random variable called the zone system = *m*. The zone system is represented by the likelihood of being in a particular zone. It is a discrete Markovian stochastic process, with discrete time determining the realization of its state. The transfer probability from zone system *i* to zone system *j* is:

$$p_{ij}=p_{r}(S_{t+1}=j|S_{t}=i),\sum_{j=1}^{m}p_{ij}=1,\forall i,j\in \left\{1,2,3,\ldots ,m\right\}$$ (7)

In this formulate (7), *m* represents the number of district systems, this study by analyzing and observing the monthly data fluctuations of pig prices; pig price fluctuations can be divided into "price decline", "price stability", and "price increase" three district system state, that is, *m* = 3, *S*_*t*_ = {1, 2, 3, *m*}, then the transfer probability matrix is:

$$\left[\begin{array}{ccc} p_{11} & p_{12} & p_{13}\\ p_{21} & p_{22} & p_{23}\\ p_{31} & p_{32} & p_{33} \end{array}\right]$$ (8)

Among this, *p*_*i*1_ + *p*_*i*2_ + *p*_*i*3_ = 1.

Then, the expression of the MSVAR model with p-order lag and including m regimes is:

$$Y_{t}=c(S_{t})+A_{1}(S_{t})(Y_{t-1})+\ldots +A_{p}(S_{t})(Y_{t-p})+u_{t}$$ (9)

Where *S*_*t*_ denotes the unobservable regime variable, matrix *v*(*S*_*t*_) denotes the intercept term associated with the zone system, and matrix *A*_*t*_(*S*_*t*_)…*A*_*p*_(*S*_*t*_) denotes the coefficients of the lagged values of the individual variables under different zone systems.

### 3.2 Data

#### 3.2.1 Explained variable

This study selects pig price as the dependent variable. Pig prices generally refer to the transaction prices of live pigs in the market, reflecting the supply-demand relationship and pig production costs. They directly influence the profitability of the pig farming industry, which affects the sustainable development of the farming sector. Pig prices exhibit characteristics such as market sensitivity, volatility, and seasonality and are influenced by various factors, including market demand, pig inventory levels, feed costs, and policy regulations. Pig price data can be directly obtained from the Brick Agricultural Database. The sample interval selected for this study spans from February 2009 to December 2022.

#### 3.2.2 Explanatory variable

In the process of an in-depth study of the existing literature on the causes of pig price fluctuation, this study systematically evaluates a variety of variables that may affect the fluctuation of pig prices and divides them into four categories: supply, demand, macroeconomic environment, and uncertainty. The following is a detailed description of the data selection process in various factors:

Uncertainty: Regarding the impact of uncertainty on the pig market, we mainly consider the economic policy uncertainty and the pig epidemics. To measure the severity of pig epidemics, we chose the width index of the pig epidemic to measure the pig epidemic, and the data comes from the Brick Agricultural Database. We use the global economic policy uncertainty index to measure economic policy uncertainty, and the data comes from the website of economic policy uncertainty (http://www.policyuncertainty.com/).

Supply: We considered multiple key variables when selecting data for supply factors. Piglet price, serving as the frontend price in the pig production chain, is sourced from the Brilliance Agricultural Data Platform. The hog inventory and pig slaughter volume, which are significant indicators reflecting the supply status of pigs, are also sourced from the Brick Agricultural Database. Pork imports can reflect the influence of the international market on the Chinese market, and the relevant data are also sourced from the Brick Agricultural Database. Pig-grain Ratio, an essential indicator for measuring the profitability of pig farming, is derived from comparing pig prices and grain prices (such as corn and wheat). These price data are sourced from the Brick Agricultural Database. In terms of costs, we considered pig farming costs, feed costs (including corn price, wheat price, and soybean meal price), and other costs for pigs. The data on these costs are sourced from the Brick Agricultural Database. Additionally, we incorporated the profit factor earned by the pig industry, including pig farming profit, pig breeding profit, pig slaughter circulation profit, and pig wholesale and retail profit. These data were collected through industry reports and databases and are directly accessible from the Brick Agricultural Database.

Demand: Regarding demand factors, we primarily consider pork exports, pork prices, prices of pork substitutes, consumer behaviors, and price levels. Pork exports constitute the terminal consumption segment of the pig industry, measured by the total tons of pork exports summarized in the export volume data published by the General Administration of Customs. Relevant data can be directly obtained from the Brick Agricultural Database. Pork substitutes primarily include chicken, mutton, beef, and eggs, for which price data can also be directly sourced from the Brick Agricultural Database. Regarding consumer behaviors, we consider the consumer expectation index, consumer satisfaction index, and consumer confidence index, which are accessible through the Qianzhan Data Network. Regarding price levels, we utilize the consumer price index (CPI) and the retail price index as metrics, with data sourced from the National Bureau of Statistics.

Macroeconomic environment: The selection of macroeconomic environment factors includes the business index of macroeconomics, the agricultural and sideline products purchasing price index, the real effective exchange rate index and the money supply (including M0, M1 and M2). These data reflect the impact of macroeconomic conditions on the pig price, and their data come from the Qianzhan Data Network.

Table 1 below provides a detailed enumeration of the indicator system for four influencing factors of pig price fluctuations, as well as their corresponding sub-variables.

**Table 1:** Influencing factors of pig price fluctuation.

Primary Indicator	Secondary Indicators	Primary Indicator	Secondary Indicators
**Uncertainty**	GEPU	**Demand**	Pork Exports
	Pig Epidemic Width Index		Chicken Market Price
**Supply**	Pig Inventory		Pork Price
	Pork Imports		Lamb Market Price
	Pig Slaughtering Volume		Beef Market Price
	Pig Farming Costs		Egg Market Price
	Pig Feed Costs		Consumer Expectation Index
	Other Costs for pig		Consumer Satisfaction Index
	Soybean Meal Price		Consumer Confidence Index
	Corn Price		CPI
	Wheat Price		Retail Price Index
	Piglet Price	**Macroeconomic Environment**	Business Index of Macroeconomics
	Pig Farming Profit		Agricultural and Sideline Products Purchasing Price Index
	Pig Breeding Profit		Real Effective Exchange Rate Index
	Pig Slaughter Circulation Profit		M0 Supply
	Pig Wholesale and Retail Profit		M1 Supply
	Pig-grain Ratio		M2 Supply

#### 3.2.3 Factor analysis

Based on the above analysis, it can be seen that pig price fluctuates due to many factors, such as supply and demand. However, if all the influencing factors are brought into the model for simulation, it will not only reduce the accuracy of the model, but will also cause multiple severe covariance problems. Therefore, the study adopts the factor analysis method to deal with the above factors affecting the pig price. Factor analysis is to extract the indicators with representative factors from multiple indicators or factors after calculating the internal structure of the correlation coefficient matrix to realize the simplicity of analyzing the indicators through dimensionality reduction [63]. Factor analysis is a useful tool for avoiding the drawbacks of subjective attribution. It also allows for evaluating the suitability of chosen indicators, ensuring the logic and scientific nature of weight allocation to some extent.

As there are many indicators in the three influencing factors of supply, demand, and macro-environment, all the indicators in the above three factors are input into SPSS software. Because the measurement standards of various influencing factors are different, the magnitude difference will be significant, so it is necessary to standardize the original data and convert it into dimensionless index evaluation values before principal component analysis. Standardization processing can be done directly in SPSS software. After that, the factor analysis was conducted, and the results of its validity test showed that the KMO statistic of all factor analyses was more significant than the minimum standard of 0.5, and the Bartlett’s spherical test rejected the original hypothesis of the unit-correlation array, which indicated that the data set in Table 1 was suitable for factor analysis. Five principal factors were extracted from the set of influencing factor information through factor analysis, with a cumulative variance contribution of 85.66% and good structural validity. Among them, the first main factor *X*_1_ contains pig breeding profit, pig farming profit, pig wholesale and retail profit, pig slaughtering volume, pig-grain ratio, pig inventory, pork imports, piglet price, and pork price; it can be summarized as profits earned by the pig industry. The second main factor *X*_2_ contains egg market price, wheat price, absolute effective exchange rate index, lamb market, beef market price, other costs of pigs, M2 supply, and white chicken market price, it can be summarized as price of alternatives. The third main factor, *X*_3_, focuses on the consumer satisfaction index, consumer confidence index, consumer expectation index, and pork exports; it can be summarized as consumer behaviors. The fourth main factor *X*_4_ includes pig feed costs, pig farming costs, corn price, soybean meal price, and the business index of macroeconomics, it can be summarized as pig farming costs. And the fifth principal factor *X*_5_ contains retail price index and the agricultural and sideline products purchasing price index, it can be summarized as change in the macroeconomic environment.

To ensure the applicability, accuracy, and reliability of the MS-VAR model, it is crucial to implement rigorous testing steps before incorporating time series data into the model construction process. The following are detailed inspection methods and corresponding results:

## 4. Results

### 4.1 Descriptive test

In order to accurately portray the impact of changes in the influencing factors on the volatility of pig prices, this part of the study first calculates the volatility of pig prices and the volatility of each influencing factor. Then it is brought into the model for analysis. The descriptive test results in Table 2 show that the JB normality test for all variables rejects the original hypothesis that volatility is normally distributed at the 5% significance level. The skewness of pig price volatility (Y) is greater than 0. The kurtosis is greater than 3, indicating that its distribution is right skewed and has the characteristics of the thick tail of the tip. Observing the median, maximum, and minimum pig price volatility shows that the pig price in the sample period has an obvious price fluctuation range, fluctuating up and down around the equilibrium price. The volatility of all kinds of influencing factors on the fluctuation of the price of pigs has obvious changes.

**Table 2:** Results of descriptive test.

	Y	Width	GEPU	*X*_1_	*X*_2_	*X*_3_	*X*_4_	*X*_5_
**mean**	100.7138	100.4999	102.0797	111.1701	109.9067	85.4188	124.9967	135.2744
**median**	100.1900	100.0000	98.4200	104.4444	95.6376	105.6962	95.2703	111.4943
**Maximum**	136.0900	222.2200	183.4900	1100.000	4600.000	3000.000	5700.000	2233.333
**Minimum**	78.0900	41.8200	63.3100	-600.0000	-1700.000	-2550.000	-2500.000	-1233.333
**Std. Dev.**	9.7893	19.7174	19.1735	127.1985	426.3024	401.2649	496.5527	293.0126
**Skewness**	0.7647	1.2298	1.2199	1.6076	6.3415	-0.9031	7.7714	3.4092
**Kurtosis**	4.8011	11.0055	6.0312	31.6224	79.3216	38.7563	102.3428	28.7549
**Jarque-Bera**	38.3836	482.1955	104.0978	5703.337	41152.69	8812.202	69510.21	4879.921
**Probability**	0.0000	0.0000	0.0000	0.0000	0.0000	0.0000	0.0000	0.0000
**Sum**	16617.77	16582.49	16843.15	18343.07	18134.60	14094.10	20624.46	22320.27
**Sum Sq. Dev.**	15716.12	63759.52	60289.93	2653433	29804327	26406218	40436591	14080448
**Observations**	165	165	165	165	165	165	165	165

### 4.2 Correlation analysis

Based on the results of the correlation analysis (as shown in Table 3), it can be observed that there exists a significant correlation between pig price and pig epidemic(Width) at a 5% significance level. Concurrently, pig prices also exhibit significant correlations with the Global Economic Policy Uncertainty Index (GEPU) and the price volatility of alternatives (*X*_2_), consumer behavior (*X*_3_), the volatility of pig farming costs (*X*_4_), and macro-environmental changes (*X*_5_) at a 10% significance level. Notably, pig prices demonstrate a high degree of significance with the volatility of profit earned by the pig industry (*X*_1_) at a 1% significance level. This finding indicates that all the identified influencing factors in this study have passed the statistical criteria for correlation testing, thereby laying a solid foundation for subsequent in-depth analysis of the impact effects of these factors on pig prices. Accordingly, we can select a regression model to advance further the exploration of the specific mechanisms through which these factors influence pig prices and the extent of their impact.

**Table 3:** Correlation coefficient table of pig price volatility(Y).

Y	Width	GEPU	*X*_1_	*X*_2_	*X*_3_	*X*_4_	*X*_5_
**Correlation**	0.1801**	0.1424*	0.2401***	0.1062*	-0.0702*	-0.1465*	0.1489*
**Probability**	0.0206	0.0681	0.0019	0.0744	0.0719	0.0604	0.0563

Note:

*, **, *** describes the significance levels of 1%, 5%, and 10%, respectively.

### 4.3 Unit root testing

To prevent false or misleading results, it is essential to assess the consistency and lack of irregularities in time series data. This research used the ADF (Augmented Dickey-Fuller) test [64] and PP (Phillips and Perron) test [65] to assess the smoothness of variables, respectively. The results are shown in Table 4 below. Based on the test findings, it is evident that all of the data successfully passed the smoothness test at a significance level of 5%. Additionally, the time series data exhibit stationarity.

**Table 4:** Results of PP test and ADF test.

Variables	PP Values	5%	P	Stability	ADF Values	5%	P	Stability
**Y**	-8.1171	-2.8789	0.0000	Steady	-8.5862	-2.8789	0.0000	Steady
**Width**	-13.9400	-2.8789	0.0000	Steady	-13.9523	-2.8789	0.0000	Steady
**GEPU**	-17.6818	-2.8789	0.0000	Steady	-15.8109	-2.8789	0.0000	Steady
*X*_1_	-9.3934	-1.9428	0.0000	Steady	-8.2849	-2.8795	0.0000	Steady
*X*_2_	-11.8279	-1.9428	0.0000	Steady	-11.7957	-1.9428	0.0000	Steady
*X*_3_	-12.6800	-2.5792	0.0000	Steady	-12.6526	-1.9428	0.0000	Steady
*X*_4_	-12.1756	-1.9428	0.0052	Steady	-12.1213	-1.9428	0.0052	Steady
*X*_5_	-12.1399	-1.9428	0.0000	Steady	-11.3776	-1.9428	0.0000	Steady

### 4.4 Multiple breakpoint testing

Various factors, including economic cycles, policy adjustments, and market changes, often influence time series data. These factors may lead to significant structural changes in the data at different time points. Breakpoint testing can identify these structural change points, namely, abrupt points or turning points within the time series. The MS-VAR model is a model that can handle the dynamic relationships of time series data under different regimes. If unrecognized breakpoints, i.e., structural change points, exist in the data, direct model construction may result in inaccurate model outcomes. Through breakpoint testing, the presence of breakpoints can be identified, and these breakpoints can be addressed, thereby avoiding the impact of structural changes on the model and enhancing the accuracy and reliability of model regression. The results of multiple breakpoint testing conducted using Eviews 9.0 are presented in Table 5 below. According to both the Schwarz Criterion and the LWZ Criterion, the number of breakpoints identified is 0, indicating that there are no significant structural change points or turning points in the data series of this study. Therefore, the model is applicable throughout the entire data range, and segmentation or adjustment is unnecessary.

**Table 5:** Results of multiple breakpoint test.

Breaks	#of Coefs.	Sum of Sq. Resids.	Log-L	Schwarz* Criterion	LWZ* Criterion
0	8	11053.58	-584.0211	4.4449*	4.6912*
1	17	8811.647	-565.2067	4.4954	5.0219
2	26	8032.165	-557.5192	4.6799	5.4903
3	35	7622.881	-553.1783	4.9048	6.0031
4	44	6785.019	-543.5140	5.0655	6.4566
5	53	6509.288	-540.0706	5.3011	6.9904
**Schwarz Criterion selected breaks: 0** **LWZ Criterion selected breaks: 0**					

Note: Minimum information criterion values displayed with *

### 4.5 Cointegration testing

Before constructing the model, this part employs the Johansen cointegration test and utilizes multivariate equation technology to examine if a long-term equilibrium connection exists among numerous variables to prevent false regression. This study uses the Eviews 9.0 program to perform a Johansen cointegration test on eight variables that are originally non-stationary series. The findings are shown in Table 6.

**Table 6:** Results of Johansen cointegration test.

Hypothesized No. of CE(s)	Eigenvalue	Trace Statistic	0.05 Critic Value	Prob.**
**None** *	0.4462	387.0835	143.6691	0.0000
**At most 1** *	0.3304	293.0995	111.7805	0.0000
**At most 2** *	0.3050	229.3230	83.93712	0.0000
**At most 3** *	0.2930	171.4522	60.06141	0.0000
**At most 4** *	0.2527	116.3096	40.17493	0.0000
**At most 5** *	0.2180	69.9751	24.27596	0.0000
**At most 6** *	0.1756	30.8695	12.32090	0.0000
**At most 7**	0.0010	0.1594	4.129906	0.7413

Note:

* display the rejections of the 0.05 level hypotheses;

** displays the MacKinnon-Haug-Michelis (1999) prob. values.

The results in Table 6 show that the p-value of at most 7 co-integration vectors is greater than 0.05, which agrees with the original hypothesis that there are at most 7 co-integration relationships. It can be seen that there is a co-integration relationship among Y, Width, GEPU, *X*_1_, *X*_2_, *X*_3_, *X*_4_ and *X*_5_, that is, there is a long-term stable equilibrium relationship between these eight variables.

### 4.6 Empirical model selection

Prior to creating the MS-VAR model, it is essential to ascertain the optimum lag number of the variables in the model. This may be achieved by selecting the ideal lag sequence of the model using the technique for establishing the optimal lag number of the general VAR model. Considering the AIC value, SC value, and HQ value of the lag test from the 1st to the 8th period, the model’s optimum lag number is 3 based on the majority rule concept.

From Krolzig’s findings [66], it can be seen that in the structural vector autoregressive model, the MSI(K)-VAR(L) model structure in which the structural term follows the state shift and the MSM(K)-VAR(L) model structure in which the mean term follows the state shift can satisfy the needs of different model settings, and the variance term can be added to follow the change of the zonal shift if necessary, i.e., the model MSMH(K)-VAR(L) model structure. The optimal structure of the model is usually based on the AIC criterion, HQ criterion, SC criterion, and the log-likelihood value of the comprehensive judgment to select the optimal model structure for analysis. Following the above principles, the analytical model of pig price influencing factors is determined as MSI(3)-VAR(3), and the processing of the model is mainly completed with the help of OX software.

### 4.7 Results of zone shift in pig price volatility

**Fig 2:**
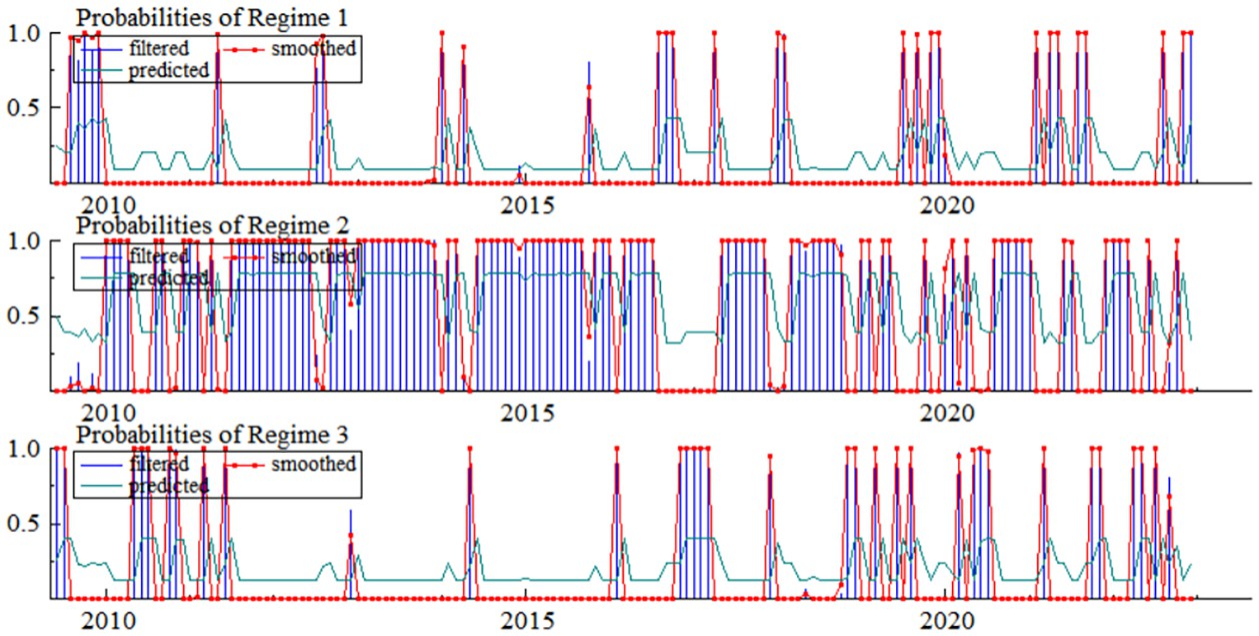
Probability plot of zone transition for pig price volatility.

After an in-depth analysis of the MSI(3)-VAR(3) model, we obtained a three-regime state transition probability graph for pig price fluctuations. These three regimes are defined as: "Pig Price Decline Phase" (Regime 1), "Pig Price Stability Phase" (Regime 2), and "Pig Price Increase Phase" (Regime 3). Through the detailed presentations in Fig 2 and Table 7, we can clearly observe that the pig market price fluctuations in China exhibit significant regime-switching characteristics.

**Table 7:** MSI(3)-VAR(3) zone system state transfer probabilities and zone system characteristics.

	Probability of a systematic transfer of regimes			Regime characteristics		
	Regime 1	Regime 2	Regime 3	sample size	proportionality	Duration(months)
**Regime 1**	0.4290	0.3259	0.2451	28.6	0.1772	1.75
**Regime 2**	0.0958	0.7799	0.1244	101.3	0.6212	4.54
**Regime 3**	0.2069	0.3919	0.4012	33.1	0.2015	1.67

For most of the period between 2010 and 2017, pig prices predominantly remained in the "Price Stability Phase" (Regime 2). The probability of this phase was as high as 0.6212, and it had the longest duration of 4.54 months. Furthermore, the transition probability from Regime 2 to itself was also extremely high, reaching 0.7799. These observations collectively suggest that the pig market environment was relatively stable and free from significant shocks during this period, thereby maintaining a relatively stable trend in pig prices.

However, from 2017 to 2022, pig price entered a non-stationary phase. As observed in Fig 2, during this period, the duration of the "Pig Price Decline Phase (Regime 1)" and the "Pig Price Increase Phase (Regime 3)" were relatively short and discontinuous, with pig prices frequently transitioning between these two regimes. The primary reasons for this change can be attributed to the outbreak of African swine fever after 2018, which led to a sharp increase in pig mortality rates and subsequent panic selling by farmers. Additionally, the widespread increase in international agricultural commodity prices also transmitted to the Chinese market, significantly elevating the cost of pig feed. Furthermore, factors such as trade frictions and changes in the macroeconomic environment further exacerbated the imbalance between supply and demand in the pig market, resulting in continuous increases and violent fluctuations in hog price.

Despite the significant fluctuations in pig prices during non-stationary periods, Table 7 reveals intriguing insights. Specifically, pig prices persist in Regime 1 (declining phase) for 1.75 months and in Regime 3 (rising phase) for 1.67 months. More importantly, the transition probability from Regime 1 to Regime 3 is 0.2451, while the likelihood of shifting from Regime 3 to Regime 1 is 0.2069. These relatively short durations and modest probability values indicate that pig prices do not readily transition directly from the rising phase to the declining phase or vice versa. In other words, there typically exists a relatively stable buffering phase between price increases and decreases in pig price, which, to some extent, mitigates sustained sharp fluctuations in prices.

### 4.8 Results of impulse response effect of factors affecting pig price

**Fig 3:**
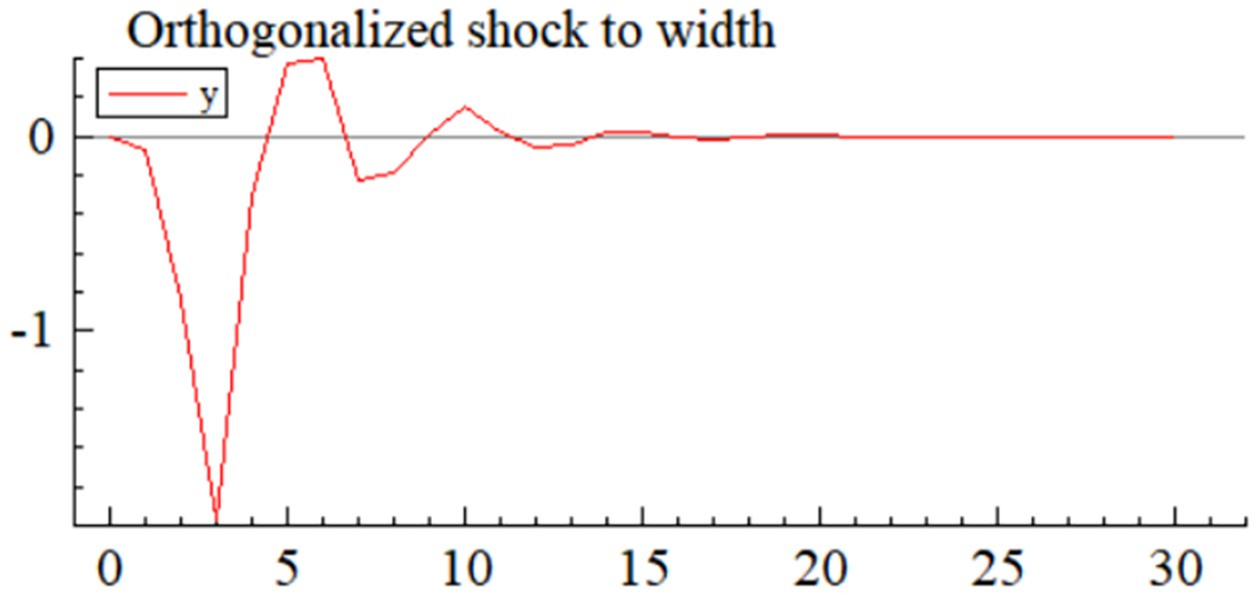
Impulse response diagram of pig epidemic situation to pig price in China.

**Fig 4:**
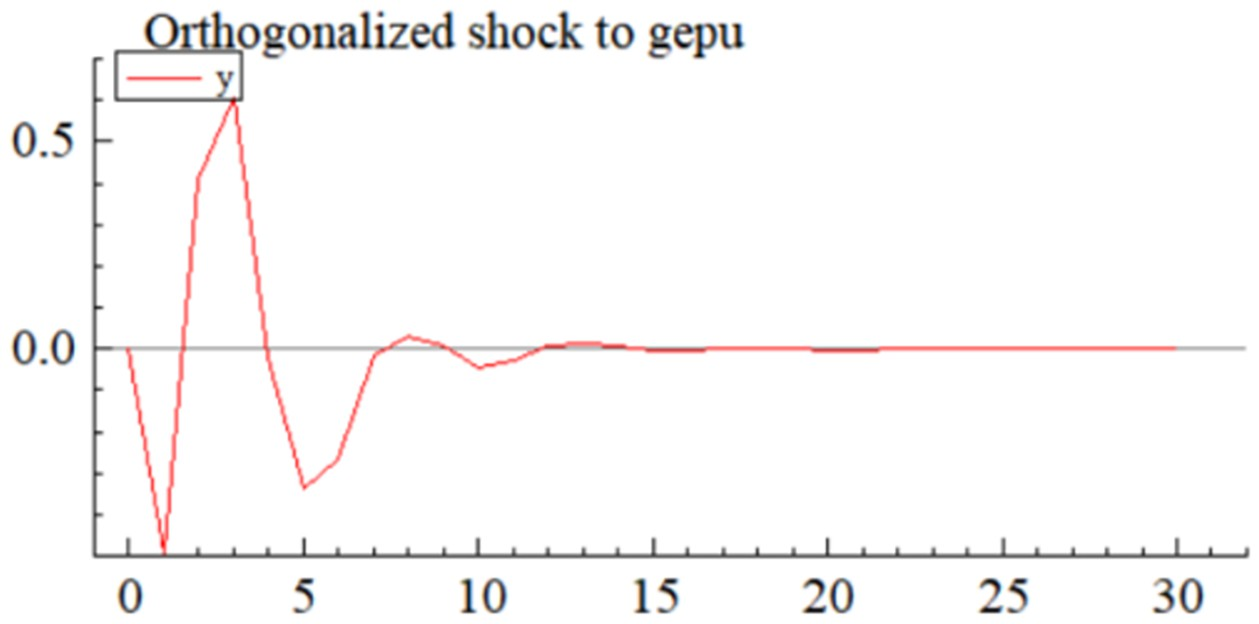
Impulse response diagram of global economic policy uncertainty index to pig price in China.

**Fig 5:**
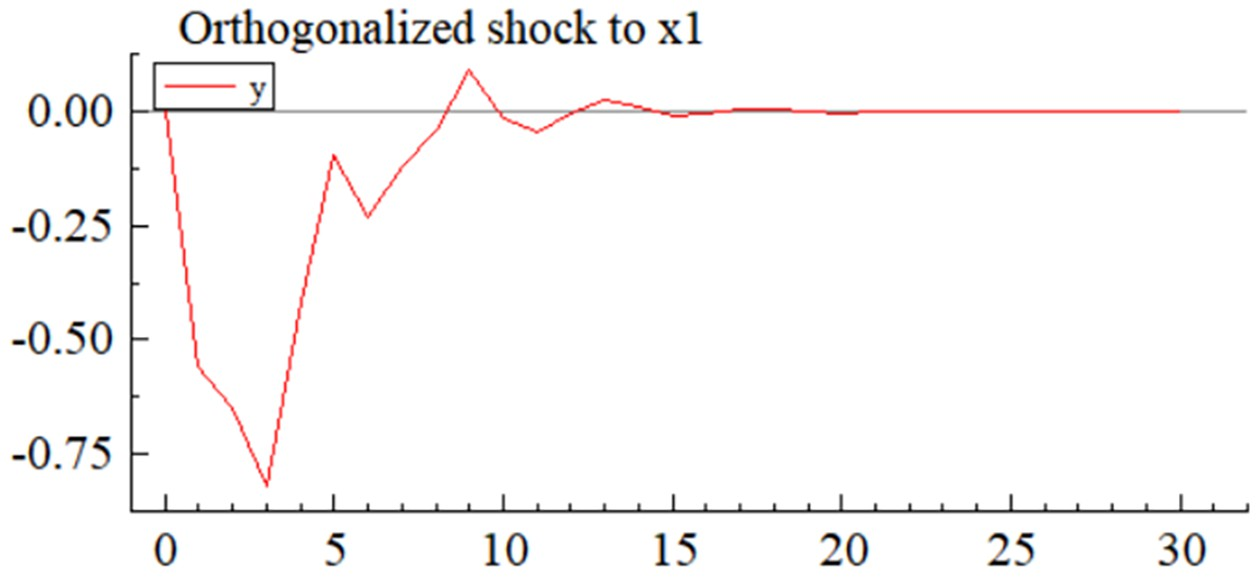
Impulse response diagram of volatility of profits earned by the pig industry to pig price in China.

**Fig 6:**
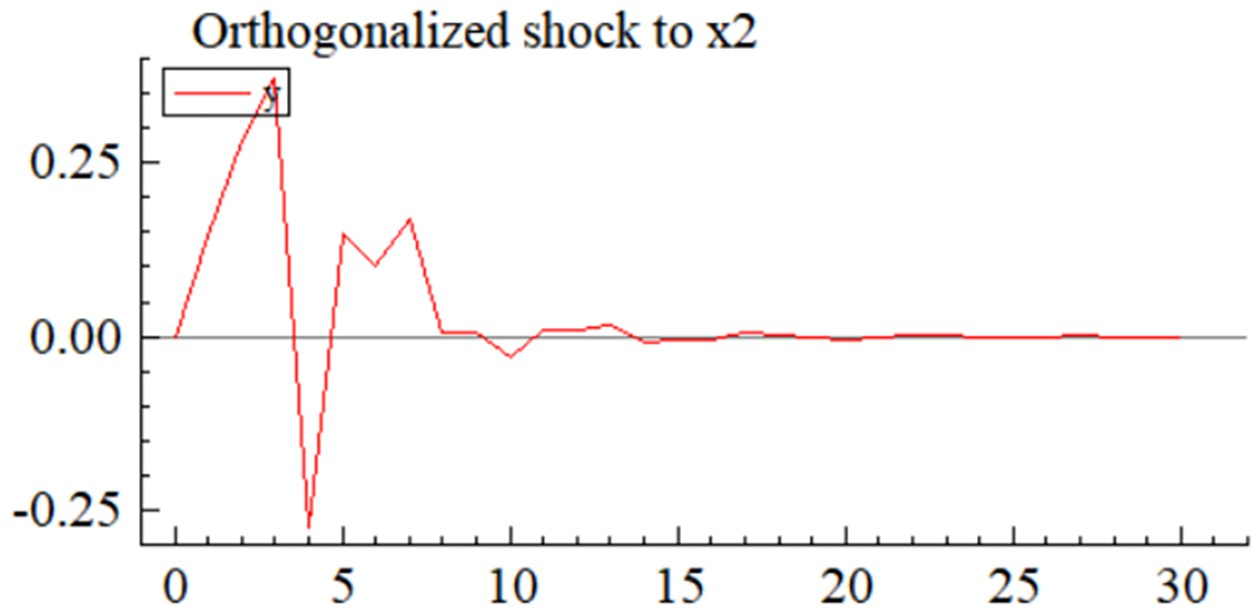
Impulse response diagram of price volatility of alternatives to pig price in China.

**Fig 7:**
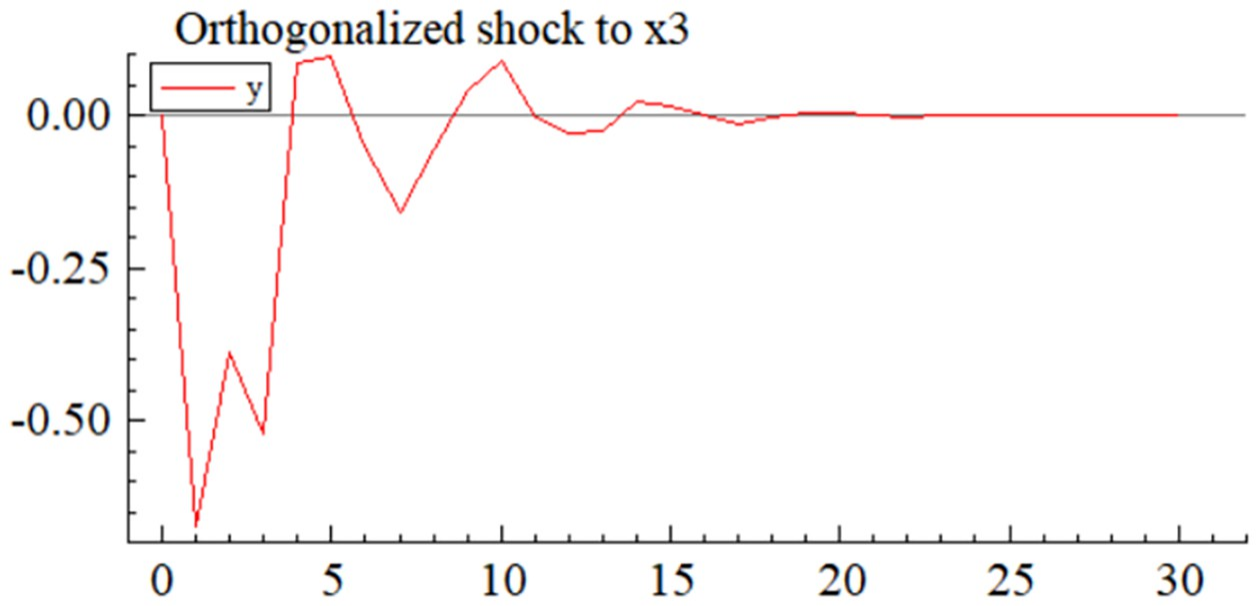
Impulse response diagram of volatility of consumer behaviors to pig price in China.

**Fig 8:**
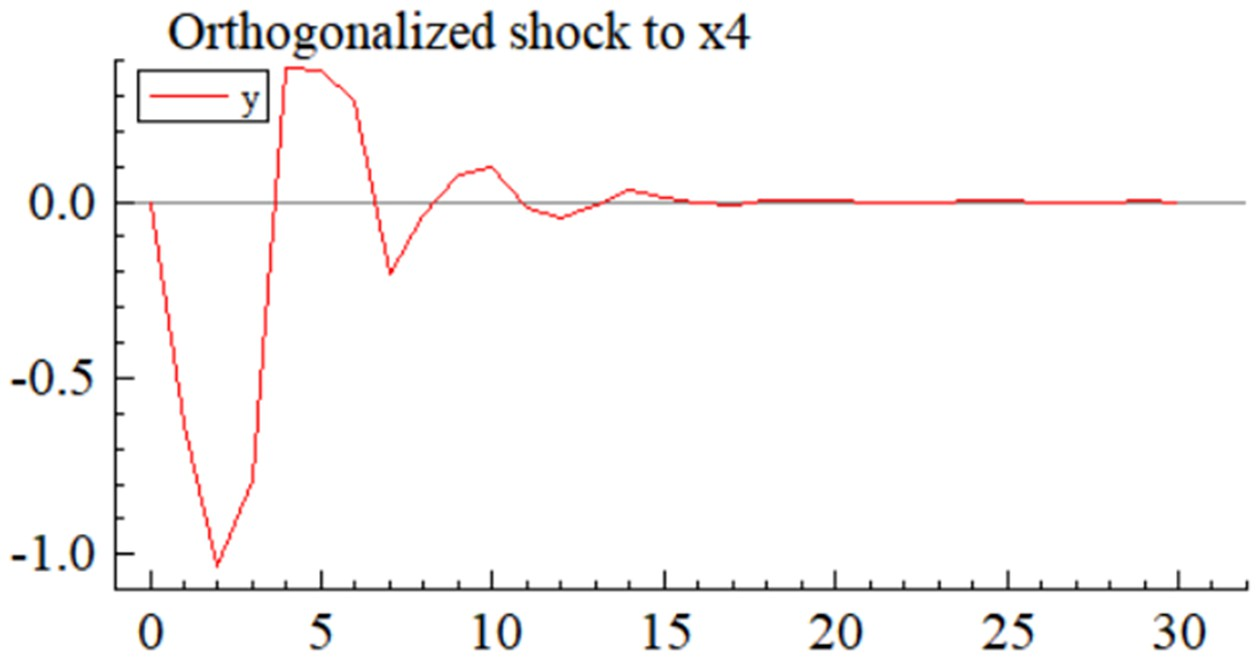
Impulse response diagram of volatility of pig farming costs to pig price in China.

**Fig 9:**
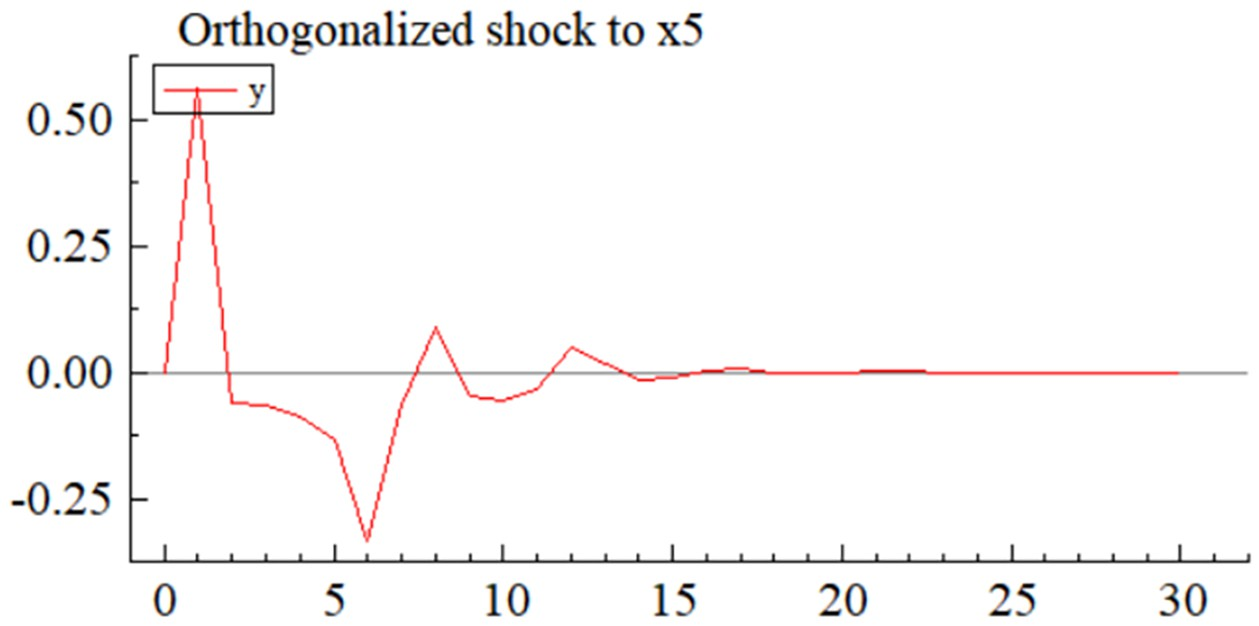
Impulse response diagram of rate of change in the macroeconomic environment to pig price in China.

**Fig 10:**
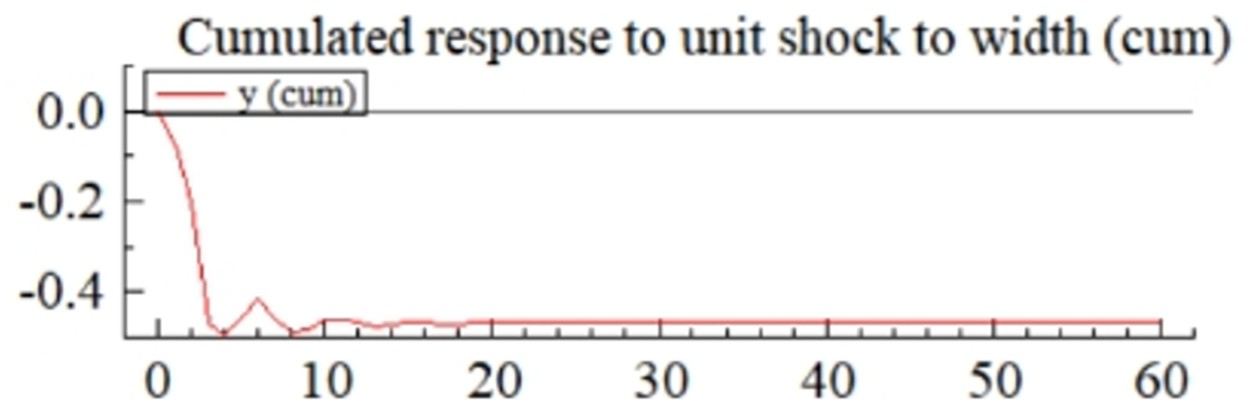
Cumulated impulse response diagram of pig epidemic situation to pig price in China.

**Fig 11:**
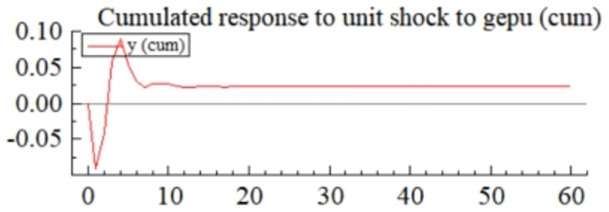
Cumulated impulse response diagram of global economic policy uncertainty index to pig price in China.

**Fig 12:**
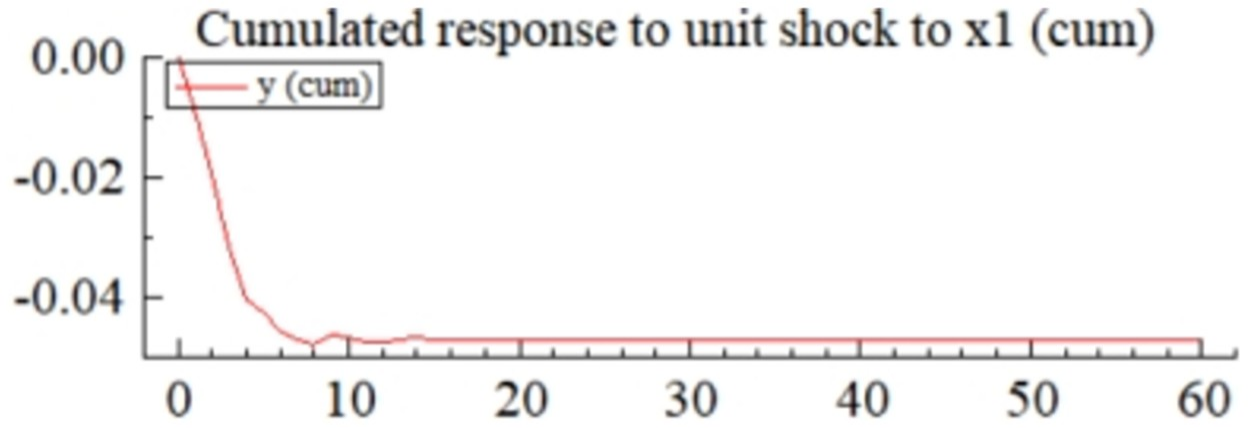
Cumulated impulse response diagram of volatility of profits earned by the pig industry to pig price in China.

**Fig 13:**
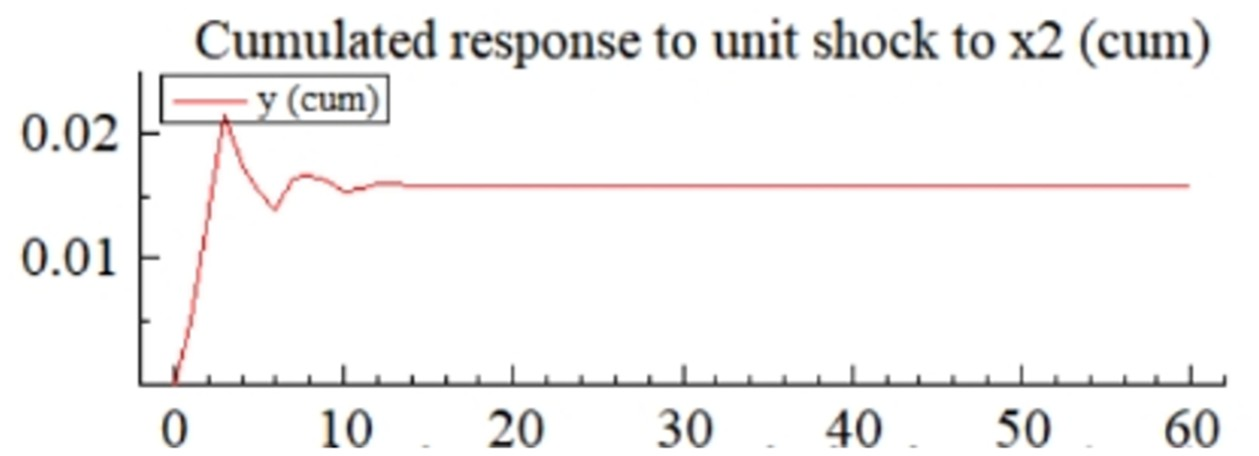
Cumulated impulse response diagram of volatility of price volatility of alternatives to pig price in China.

**Fig 14:**
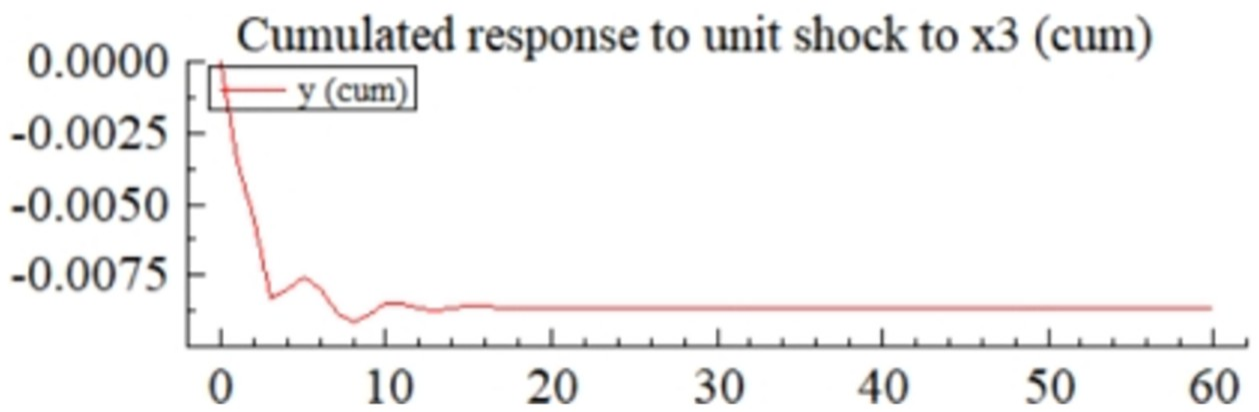
Cumulated impulse response diagram of volatility of consumer behaviors to pig price in China.

**Fig 15:**
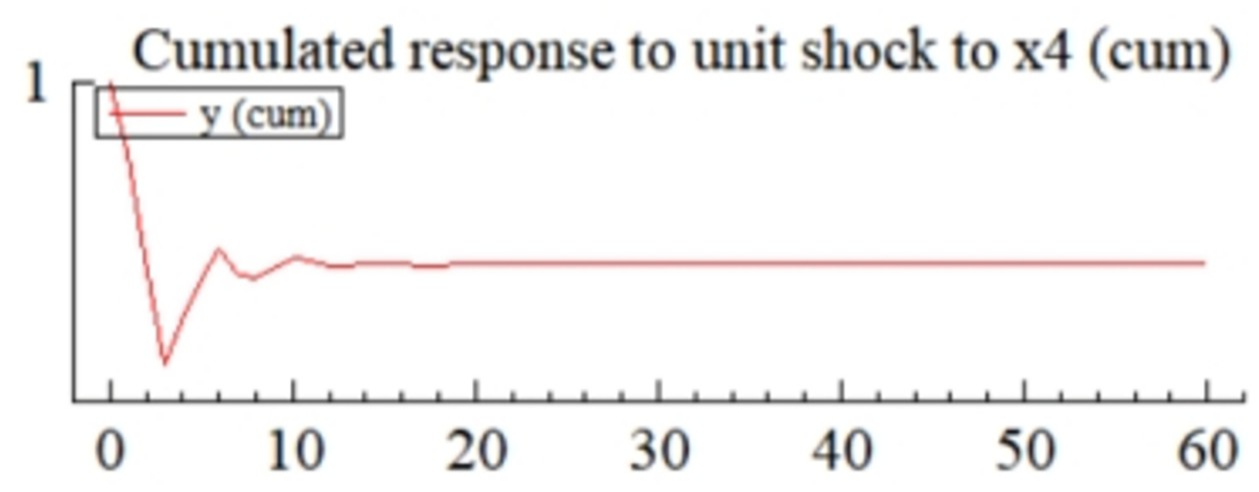
Cumulated impulse response diagram of volatility of pig farming costs to pig price in China.

**Fig 16:**
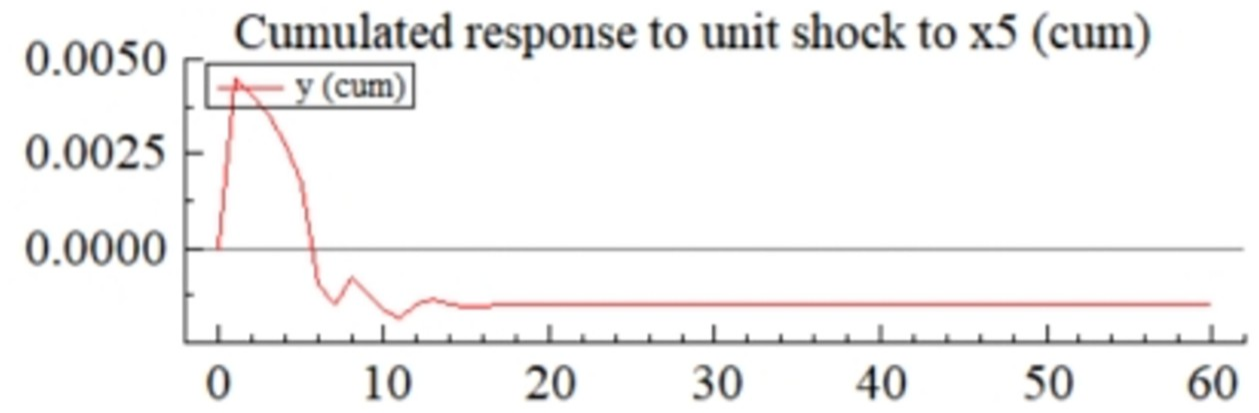
Cumulated impulse response diagram of rate of change in the macroeconomic environment to pig price in China.

MSI(3) -VAR(3) structural model was utilized to analyze the impulse response plots of the effects of domestic pig price-related influencing factors on pig prices during the sample period. The impulse effects of the relevant factors on pig prices over the sample period are shown in Figs 3–9. It can be seen that a positive shock from pig epidemics leads to a larger negative response in pig prices first; and then shows a small amplitude of positive and negative fluctuations. By comparing the impulse response diagrams of other factors and observing the cumulative impulse response effect diagram of each factor in Figs 10–16, it can be seen that the pig epidemic has brought the deepest impact on pig prices. This shows that: among all the influencing factors, the impact effect of the pig epidemic on the pig price is the largest.

## 5. Discussion

By observing the occurrence time and "pig cycle" of pig epidemics in the past, it can also be learned; the pig epidemic is the most crucial reason for the wide range of persistent fluctuations in the price of pigs, pig epidemic going round, and begin again is the main reason for the formation of the "pig cycle". As can be seen from Fig 3, the impact of the pig epidemic on pig prices in the first three periods of the response coefficient was negative and rapid growth and reached the maximum value in the third period, and then gradually turned into a positive impact until after the 20th period of the effects gradually disappeared; pig price on the cumulative impulse response to the pig epidemic also in the fourth period to reach a negative maximum, the overall impact of the pig epidemic on pig price for the negative direction (Fig 10). The extent of the effects of an outbreak on pig prices depends on factors such as the severity of the epidemic, the speed of its spread, the scale of farming, and the ability of the market to regulate it. The outbreak of pig disease at the beginning of the herd is very susceptible to infection and disease, culling and quarantine, and other measures to cause a sharp decline in the supply of pigs on the farm; at the same time, the spread of the pig epidemic will trigger panic in the market, investors and traders may be such panic through the rapid sale of existing pig futures and other means of transmission to the pig market, depressing the market price of pigs.

For consumers, the pig epidemic network attention and public opinion dissemination will reduce the consumer information on meat products and thus reduce the demand for pork products [67], further depressing the price of pigs; to the recovery period of the pig epidemic, due to the pork is the primary source of protein for the population, so the demand for pork to recover faster, and at this time due to the growth cycle of the reasons for the recovery of the supply of pigs there is a lag. At the same time, due to the producers are expected to rise in price after the pressure of the barn to sell the behavior of the market, the pig market supply and demand is imbalance, resulting in the pig price continue to rise.

A positive shock to the economic policy uncertainty index leads to positive and negative fluctuations in the impulse response (Fig 4) and cumulative impulse response (Fig 11) of the pig price, and the absolute value is not very large, which indicates that the impact of the economic policy uncertainty index on the pig price is uncertain and relatively small. The shock to pig prices is the result of a combination of multiple economic policy uncertainty events, and the impacts from different events can be positive or negative, so the results from various economic policy uncertainty event shocks can be misaligned with the timing of the shocks. In addition, events that significantly impact pig prices are not necessarily those with the most tremendous economic policy uncertainty, but must be important events that are closely related to the livestock industry and its markets. Uncertainty in the international economic and political environment will, on the one hand, be transmitted to the futures market, grain market, etc., which will lead to price fluctuations of feed grains such as soybeans, corn, etc., and indirectly be transmitted to the domestic impact effect on the price of pigs [68]; on the other hand, uncertainty in the economic policy will have a wide-ranging impact on the macroeconomic environment, including the monetary policy, GDP growth and the level of employment, etc., which will affect the level of demand and the purchasing power of the consumers, and thus have a positive impact on the price of pigs [69].

By observing Figs 3–16., we can know that among many factors affecting supply and demand, the volatility of pig farming costs (*X*_4_) significantly affects the fluctuation of pig market price. The impulse response diagram (Fig 8) intuitively shows that after *X*_4_ suffered a positive impact, the pig price showed a more intense positive and negative fluctuation trend. Further investigation of the cumulated impulse response diagram (Fig 15) shows that the pig price generally presents the characteristics of a positive response to this shock, which reaches its peak in the fourth period, then gradually weakens, and tends to a stable state after the sixteenth period. This series of dynamic changes fully shows that the volatility of pig farming costs has a far-reaching impact on pig price and a long-lasting effect.

Compared to various other factors, pig farming costs have exerted a more pronounced influence on pig prices, a phenomenon rooted in the combined effects at multiple levels. Firstly, the composition of pig farming costs is complex, encompassing feed costs, expenses for equipment and facilities required for farming, control costs of the farming environment, labor costs, and disease prevention and control costs, among others. Fluctuations in these cost factors are directly linked to the overall cost level of pig farming. An increase in any of these cost factors compresses the profit margins of farmers, prompting them to respond to cost pressures by raising pig sales prices. Secondly, pig farming costs are highly sensitive to changes in market prices. Feed costs, such as fluctuations in the prices of key raw materials like corn and soybeans and costs related to the farming environment and disease prevention and control, fluctuate with changes in market conditions. These fluctuations are swiftly reflected in pig prices, rendering pig prices highly responsive to changes in farming costs. Moreover, changes in farming costs also influence pig prices through the regulatory role of supply and demand. When farming costs rise, some farmers may reduce their farming scale due to narrowed profit margins, decreasing pig supply. With relatively stable demand, this reduction in supply further pushes up pig prices. Conversely, farmers may expand their farming scale and increase pig supply when farming costs decline, thereby restraining price increases. Lastly, compared to other influencing factors, such as policy regulations and international market changes, variations in pig farming costs are more frequent, significant, and direct. Although policy regulations can impact pig prices, their effects often have lags and uncertainties. While international market changes can also affect domestic pig prices through channels such as global trade, this impact is generally more indirect and slower. In contrast, changes in pig farming costs are more direct, significant, and observable, making them one of the critical factors influencing pig prices.

Among other supply and demand factors, a positive shock to the price volatility of alternatives (*X*_2_) leads to positive and negative impulse effects on pig prices (Fig 6), but positive shocks and the effects dominate the cumulative impulse effects are more pronounced than those of other factors (Fig 13). This is consistent with the previous theoretical analysis and the findings of the existing literature. The diversification of meat products makes more and more substitutes for pig products. The substitution effect becomes stronger and stronger, and the changes in pig prices due to substitutes become more complicated. Beef and mutton are much more expensive than pork, and rational consumers will buy beef and mutton only after the price of pork has been satisfied, so the substitution effect of beef and mutton on the price of pork is fragile. Chicken and pork are similarly priced, so both markets are closely related, and price transmission is faster [69]. An increase (decrease) in the price of chicken will cause the demand for pork to increase (decrease), which will drive the price of pork up (down).

The profits made by the pig industry also have an important influence on the price of pigs. From the impulse response graph (Fig 5) and cumulative impulse response graph (Fig 12) of the volatility of profit earned by the pig industry (*X*_1_) on the volatility of pig price, It is evident that a favorable influence on the profitability of the pig sector would result in both positive and negative oscillations in pig prices, with a stronger emphasis on the negative variations. By examining the cumulative impulse response chart, it is evident that the fluctuation in total profitability within the pig business has a detrimental effect on pig pricing. The primary effect is. The correlation between the profitability of breeding and pig pricing is intricate and influenced by several elements, such as supply and demand dynamics, production expenses, governmental regulations, and market demand. When pig prices stay elevated, every aspect of the pig business will become lucrative, prompting farmers to increase their pig production capacity and steadily decreasing pig pricing. Nevertheless, the increasing expenses associated with pig farming will form a foundation for pricing, preventing them from continuously declining.

Observing the impulse response effect graph of consumer behavior (*X*_3_) on pig price, it can be seen that the pig price has a large amplitude of positive and negative fluctuations after a positive impact of consumer behavior (Fig 7). The cumulative impulse response graph shows that the overall changes in consumer behavior will negatively affect the volatility of pig prices, and it reaches the maximum value in the 9th period (Fig 14). The impact tends to be stable after the 16th period. There is a complex interaction between consumer behavior and pig prices. For example, pork prices have been volatile recently due to external factors such as African swine fever, environmental regulations, and new crown epidemics. These factors will not only affect the supply of pigs. Still, they will also make consumers more concerned about food safety, changing their purchasing behavior and consumption habits and further exacerbating the volatility of pig prices.

From Fig 9, it can be seen that a positive shock of macro-environmental changes leads to large positive and negative fluctuations in pig price volatility, and the observation of the corresponding cumulative impulse response (Fig 16) also shows that the overall impact of macro-environmental changes *X*_5_ on pig price volatility also exists in the switching of positive and negative shocks, and finally stabilizes with a weaker negative impact. It can be seen that the macro-environmental fluctuations in the price of pigs also create more significant uncertainty. The effect of the macroeconomic environment on pig prices is multifaceted: for example, the speed of economic growth directly affects the income status and purchasing power of consumers, which indirectly leads to the rise and fall of pig prices; the level of the inflation rate also affects the cost of pig rearing and thus causes fluctuations in the price of pigs; when the central bank implements a loose (tightening) monetary policy, the supply of capital in the market may increase (decrease), which pushes up (pulls down) the price of pigs; the outbreak of the U.S.-China trade war in 2018–2019 led to a significant reduction in China’s imports of pork from the U.S., which led to a relative increase in the domestic demand for pork, which further pushed up the price of pigs, an event that suggests that changes in the international trade environment also affect the pig prices.

## 6. Conclusion and policy recommendations

### 6.1 Conclusion

In this study, pig price in China was selected as the main research object, and a number of key explanatory variables were comprehensively screened from four dimensions: supply, demand, macroeconomic environment, and uncertainty shock by factor analysis, and the MSI(3)-VAR(3) model was constructed. This method not only deeply analyzes the dynamic relationship among the variables from a nonlinear perspective but also accurately describes the leading factors and the formation mechanism of China’s pig price fluctuation with the help of an impulse response diagram and cumulative impulse effect diagram. This study not only enriches the academic achievements in the field of pig price fluctuations but also provides strong decision support for promoting the sustainable development of the pig industry. After detailed analysis, this study draws the following conclusions:

1. The change characteristics of the regional system of hog price fluctuation are remarkable: the research results show that the fluctuation of hog price presents obvious change characteristics of three regional systems (upward, stable, and downward), which reveals the periodic changes experienced by the hog market at different stages. Especially after the outbreak of African swine fever in China in 2018, the switching frequency of live pig prices between the upward and downward zones increased significantly, highlighting the huge impact of major epidemics on market stability.
2. The pig epidemic is the leading factor in pig price fluctuation among many internal and external factors affecting pig price; the pig epidemic situation is undoubtedly the most influential leading factor. It not only directly led to a sharp decline in the supply of live pigs but also further aggravated the fluctuation of pig prices by affecting market expectations and consumer behavior. In addition, the epidemic also indirectly triggered the intensification of the "pig cycle", making the periodic fluctuation of the pig market more frequent and intense.
3. The volatility of pig farming costs has a far-reaching impact on pig prices: from the related internal factors that affect pig prices, the change in breeding cost has the most significant impact on pig prices. The increase or decrease of breeding cost will directly affect the profit space and production decision of pig producers and then have a far-reaching impact on the balance of supply and demand and the price level of the pig market. In addition, external shocks, such as policy adjustment and market competition, mostly indirectly affect the pig price by affecting the cost of raising pigs.
4. Mechanism of pig price formation under the joint action of multiple factors: This study also found that the fluctuation of pig price cannot be entirely explained by a single factor but is the result of the joint action of multiple factors. In addition to the epidemic situation of pigs and the cost of breeding, the change in market demand, the change in the macroeconomic environment, and the impact of uncertainty have also had an important impact on pig prices. These factors interact and restrict each other, which together constitute a complex mechanism of pig price fluctuation.

### 6.2 Policy recommendations

Based on the above conclusions, this paper proposes the following relevant policy recommendations:

Attached is great importance to the prevention and control of epidemic risks, as well as adopting multi-channel and multi-methods to ensure supply and stabilize prices. From the conclusion, it is evident that within the framework of sustainable development in the pig farming industry, epidemic risk prevention and control constitute one of the core challenges. Therefore, to effectively respond to emergencies such as swine diseases, it is particularly urgent to establish a systematic and efficient risk response mechanism. Specifically, efforts should be made to set up a national-level swine epidemic emergency response center equipped with advanced monitoring equipment and technological means to ensure rapid response and the adoption of scientific and precise prevention and control measures in the event of an outbreak. A pig farming industry monitoring and early warning system should also be established, which requires robust data processing and analytical capabilities. Through in-depth cooperation with international organizations, global pig market data should be shared to enhance the accuracy and international influence of the early warning system. On this basis, a series of medium- and long-term policies need to be formulated and implemented, covering aspects such as post-epidemic capacity restoration, technological upgrading, industrial upgrading, and market regulation to ensure the stable development of the pig farming industry. Furthermore, enforcing these policies should be strengthened to ensure their effective implementation, providing a solid institutional and policy guarantee for the sustainable development of the pig farming industry.

Further, it reduces the cost of pig farming and improves the competitiveness of the pig market. To reduce pig farming costs and enhance market competitiveness, it is necessary to address the issue from two aspects: technological innovation and policy support. In terms of technological innovation, efforts should be made to introduce advanced farming technologies and equipment actively, leveraging technological advancements to improve the reproduction rate and survival rate of pigs while reducing feed consumption. Cooperation and exchanges with internationally renowned farming enterprises should be strengthened to develop efficient and low-cost feed formulations jointly. Additionally, technical training for pig farmers should be intensified to guide them in rationally formulating feed components and improving feed utilization efficiency. Furthermore, the promotion of modern, efficient, and environmentally friendly pig farming methods and the enhancement of pig farm infrastructure should be prioritized to improve labor productivity. In terms of policy support, preferential policies such as loans and subsidies should be provided to reduce the financing costs of pig farmers and encourage them to expand production scale and enhance production efficiency. Meanwhile, the government should strengthen the supervision and regulation of the pig farming industry to ensure fair competition and healthy market development. Implementing these measures will contribute to reducing pig farming costs, improving market competitiveness, and promoting the transformation, upgrading, and sustainable development of the pig farming industry.

Timely update of pig market information so farmers can adjust their pig production plans in time to avoid price risks. To promptly update pig market information for stabilizing market prices, it is imperative to enhance the efficiency of information collection and transmission while establishing an effective price stabilization mechanism. In terms of information updating, a regular update mechanism for data such as pig epidemic situations and inventory levels should be established to ensure the accuracy and timeliness of information. Additionally, the construction of an Internet of Things (IoT) for pig farming early warning should be strengthened, enabling real-time sharing and rapid response to market information through IoT technology. Based on this, market information should guide farmers in adjusting their pig production plans, thereby avoiding market supply-demand imbalances caused by blind production. In terms of constructing a price stabilization mechanism, a pig price monitoring and early warning system should be established to detect abnormal price fluctuations promptly and take corresponding regulatory measures. Meanwhile, the government should also stabilize market price fluctuations through measures such as releasing government-held pork reserves and regulating pork imports. Furthermore, cooperation and exchanges with other countries and regions should be strengthened to jointly address the challenges and opportunities in the global pig market. The implementation of these measures will help reduce the probability of market supply-demand imbalances, improve the transparency and stability of the pig market, and provide robust support for the sustainable development of the pig farming industry.

## 7. Limitations and future directions

Last, but not least, we acknowledge several limitations in our study that warrant attention. While we have endeavored to consider as many factors influencing pig price as possible in our data selection, it is undeniable that the dynamics of the pork market are exceedingly complex. Several crucial factors, such as the volume of pork reserves held by the state and relevant governmental policies, remain unaccounted for in this paper. This omission stems from the practical difficulties of obtaining or quantifying such data. Despite their potential significance, these factors pose substantial challenges in terms of accessibility and measurement, thereby limiting their inclusion in our analysis.

Furthermore, our study primarily focuses on identifying the causes of pig price fluctuations and pinpointing the dominant factors that drive abnormal volatility. However, we have not delved deeper into the intricate mechanisms through which these factors influence pig price volatility. This gap in our analysis underscores the need for future research to explore these mechanisms in greater detail. Discussing these issues necessitates a more comprehensive examination, potentially incorporating advanced statistical techniques or economic modeling, to fully elucidate the pathways through which various factors impact pig price.

In light of these limitations, we emphasize the importance of continued research endeavors to build upon our findings. Future studies should strive to address these limitations by exploring the excluded factors and delving into the underlying mechanisms that govern pig price dynamics. By doing so, researchers can contribute to a more comprehensive and nuanced understanding of the pork market, ultimately facilitating better-informed decision-making and policy formulation in this critical agricultural sector.
